# Expression Profiles of ASIC1/2 and TRPV1/4 in Common Skin Tumors

**DOI:** 10.3390/ijms22116024

**Published:** 2021-06-02

**Authors:** Kirsten Ackermann, Susanne Wallner, Christoph Brochhausen, Stephan Schreml

**Affiliations:** 1 Department of Dermatology, University Medical Center Regensburg, 93053 Regensburg, Germany; kirsten.ackermann@gmx.net (K.A.); susanne.wallner@ukr.de (S.W.); 2 Institute of Pathology, University of Regensburg, 93053 Regensburg, Germany; christoph.brochhausen@ukr.de

**Keywords:** melanoma, squamous cell carcinoma, basal cell carcinoma, proton-sensitive ion channels

## Abstract

The acid-sensing ion channels ASIC1 and ASIC2, as well as the transient receptor potential vanilloid channels TRPV1 and TRPV4, are proton-gated cation channels that can be activated by low extracellular pH (pH_e_), which is a hallmark of the tumor microenvironment in solid tumors. However, the role of these channels in the development of skin tumors is still unclear. In this study, we investigated the expression profiles of ASIC1, ASIC2, TRPV1 and TRPV4 in malignant melanoma (MM), squamous cell carcinoma (SCC), basal cell carcinoma (BCC) and in nevus cell nevi (NCN). We conducted immunohistochemistry using paraffin-embedded tissue samples from patients and found that most skin tumors express ASIC1/2 and TRPV1/4. Striking results were that BCCs are often negative for ASIC2, while nearly all SCCs express this marker. Epidermal MM sometimes seem to lack ASIC1 in contrast to NCN. Dermal portions of MM show strong expression of TRPV1 more frequently than dermal NCN portions. Some NCN show a decreasing ASIC1/2 expression in deeper dermal tumor tissue, while MM seem to not lose ASIC1/2 in deeper dermal portions. ASIC1, ASIC2, TRPV1 and TRPV4 in skin tumors might be involved in tumor progression, thus being potential diagnostic and therapeutic targets.

## 1. Introduction

Melanoma and non-melanoma skin cancers (NMSCs) are the most prevalent cancers among the white population, exhibiting an increasing incidence rate worldwide [1]. The WHO counts between 2 to 3 million new cases of NMSC per year, being 18–20 times higher than melanoma. However, due to its risk of metastasis, the malignant melanoma (MM) is responsible for 90% of deaths among skin cancers, with a yearly increasing incidence rate between 4 and 6% [2]. The group of NMSC includes basal cell carcinomas (BCCs), which account for around 80% of NMSC, and squamous cell carcinomas (SCCs), with around 20% of NMSC. Only 1% can be classified as other skin tumors [3]. Nevus cell nevi (NCN) are benign neoplasms, but about 10–30% of melanomas arise from NCN [4]. Even if the mortality rate and metastatic potential of NMSCs are low, those tumors lead to enormous morbidity and extensive costs for our health system [5]. Therefore, it is important to find new therapeutic targets in MM and NMSC for future treatments.

Tumor formation changes the physical microenvironment in the tissue. Little vascular perfusion, regional hypoxia and the subsequent anaerobic glucose metabolism lead to lactic acid and, hence, to extracellular acidosis in tumors with extracellular pH (pH_e_) as low as 6.5 [6]. Furthermore, membrane-bound transporters (monocarboxylate transporters MCTs 1–4, carboanhydrases CA2/9/12, sodium hydrogen exchanger 1 NHE, vacuolar type ATPases VATPases, sodium bicarbonate symporters) contribute to the acidified tumor microenvironment (TME) [7]. In physiological conditions, the pH_e_ is higher (7.2–7.4) than the intracellular pH_i_ (6.9–7.2), whereas in a tumor environment, the so-called reversed pH gradient (pH_e_ < pH_i_) develops [8]. This reversed pH gradient (or inside-out pH gradient) is harmful to normal cells, as cellular acidification in general leads to apoptosis. In tumor cells, however, it causes migration and invasion and, hence, benefits tumor growth [6]. In contrast to normal cells, tumor cells can adjust to survive in low pH by increasing glycolytic activity and expression of proton transporters, which stabilize intracellular pH [9]. Several of these transporters and pumps have already been detected to play a role in the maintenance of TME, such as carbonic anhydrases (CA2,CA9, CA12), V-ATPases (vacuolar-type H^+^ ATPases), Na^+^/HCO^−^ 3-Co-transporters, the monocarboxylate transporters MCT 1–4 or Na^+^/H^+^ exchanger 1 (NHE1) [10]. Through changes in their expression or activity, these plasma membrane proteins promote H^+^ efflux, thus leading to the typical alkaline pH_i_ and the acidic pH_e_ in tumor cells [10]. Cancer cells need to detect the dysregulated pH by sensors to mediate adequate cellular response. Acid-sensing proteins transmit signals to the cytoplasm and nucleus, hence influencing intracellular signal transduction pathways and gene expression [10]. One group of these sensors is the proton-sensitive G-protein coupled receptors (pH-GPCRs) [11]. We recently published first data on the expression profiles of pH-GPCRs in various skin tumors [8,12].

Other proton-sensing sensors in the plasma membrane are the transient receptor potential vanilloid channels (TRPVs) as well as the acid-sensitive ion channels (ASICs). Little is, however, known on their expression and role in skin tumors.

Transient receptor vanilloid potential ion channels (TRPVs) are a group of subfamilies numerously and diversely expressed in several tissues and organs, where they perform pleiotropic physiological and pathological functions. These nonselective cation channels were originally characterized as “polymodal cellular sensors” in neurons, being activated by chemical, physical and thermal stimuli [13]. A subgroup of these channels are the Ca^2+^-permeable, nonselective thermo-TRPs TRPV1 and TRPV4 [14]. These proton-sensing proteins are both activated by extracellular acidity [10]. Furthermore, TRPV1 is stimulated by vanilloid compounds (capsaicin and resiniferatoxin), injurious heat (≥43 °C) and some eicosanoids [15]. TRPV4 is activated by lower temperature (>24 °C) and by hypoosmotic stimulation [15]. Apart from neuronal cells, the expression of TRPV1 and TRPV4 has been proven in a wide range of tissues, amongst others in epidermal keratinocytes [16]. Moreover, they play a role in the regulation of cell apoptosis and survival by regulating calcium signaling, which is essential for the apoptosis-driven differentiation program of keratinocytes [16]. TRPV1 has been found within the skin in epidermal and hair follicle keratinocytes, dermal mast cells, in sebaceous glands and dendritic cells [15]. TRPV4 contributes additionally to cell survival after skin exposure to heat and to the control of skin permeability barrier by modulating tight junction proteins [17]. Its activation promotes barrier regeneration, which was demonstrated by the fact that an inferior epidermal barrier (e.g., untight cell–cell junctions) was found in TRPV4-deficient mice [13]. TRPV4 has been identified in basal and suprabasal keratinocytes [15].

Acid-sensing ion channels (ASICs) are cation channels that belong to the degenerin/epithelial Na^+^ channel (DEG/ENaC) superfamily and can be activated by extracellular acidification. They are mostly expressed in the central nervous system and in peripheral sensory neurons. There are seven subunits from four genes (namely ASIC1a, ASIC1b1, ASIC1b2, ASIC2a, ASIC2b, ASIC3 and ASIC4) [10]. Each subunit comprises two transmembrane domains, connected with a large extracellular cysteine-rich loop, which are trimeric assemblies. ASIC1 is Na^+^- and Ca^2+^-permeable, whereas other types of ASICs are only permeable to Na^+^ [6]. ASICs have different functions in the peripheral and central nervous system in physiological as well as in pathological processes. In the CNS, ASIC1 channels participate in neuroplasticity, regulation of fear behaviors, learning, memory functions and pain sensation [18]. ASIC2 plays a role in retinal integrity and neuronal viability in cerebral ischemia [19]. In the peripheral nervous system, they are involved in nociception and mechano-sensation [20]. More relevant for the current study, however, is the fact that these ASICs are also expressed in non-neuronal cells (e.g., keratinocytes, bone, dendritic cells, vascular smooth muscle [6]), where they contribute to pH homeostasis, cellular migration and inflammation [21].

There are a few reports about the expression and the functions of TRPVs and ASICs in other tumors [22]. Nevertheless, there is no sufficient information about their presence and function in skin tumors. In this study, we investigate the expressions of ASIC1, ASIC2, TRPV1 and TRPV4 in squamous cell carcinoma (SCC), basal cell carcinoma (BCC), malignant melanoma (MM) and in nevus cell nevi (NCN).

## 2. Results

We summarized our results of standard histological samples and TMAs with about 20–30 samples per tumor type. Figure 1, Figure 2, Figure 3 and Figure 4 show representative IHC staining results, and the other samples are depicted in the Supplementary Figures S1–S18. An overview of staining results/scores for regular IHC and TMA is shown in Figure 5. Additional TMA and scores, as well as general patient information, is given in Supplementary Figure S16 and Supplementary Tables S1–S5.

**Figure 1:**
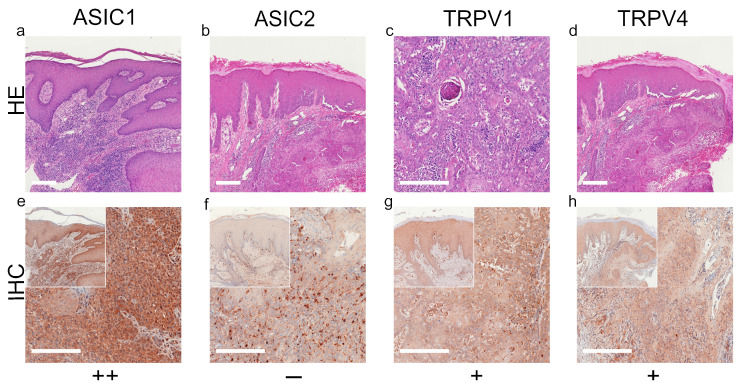
Immunohistochemistry of SCC. Immunohistochemical staining for ASIC1, ASIC2, TRPV1 and TRPV4 in SCC tissue. (**a**–**d**) H&E staining, (**e**–**h**) immunohistochemical staining, inserted smaller pictures represent a two times larger perspective. Scale bars represent 200 μm. (**a**–**h**) Patient 8. This SCC shows no expression of ASIC2, only some peritumoral lymphocytes appear positive. The tumor cells show a weak, positive expression of TRPV1 and TRPV4. ASIC1 is expressed strongly on tumor cells. For more stainings of other SCCs, see Supplementary Figures S1–S3.

**Figure 2:**
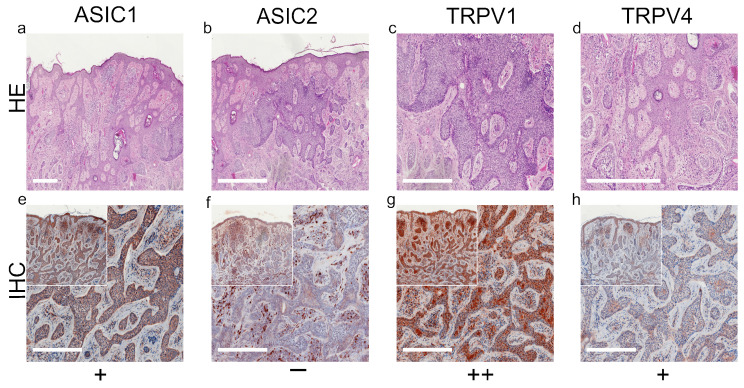
Immunohistochemistry of BCC. Immunohistochemical staining for ASIC1, ASIC2, TRPV1 and TRPV4 in BCC tissue. (**a**–**d**) H&E staining, (**e**–**h**) immunohistochemical staining, inserted smaller pictures give an overview. Scale bars represent 200 μm. (**a**–**h**) Patient 10. This BCC shows a strong expression of ASIC1 and TRPV1. The expression of TRPV4 is weak and positive, but this BCC shows no expression of ASIC2. For more stainings of other BCCs, see Supplementary Figures S4–S7.

**Figure 3:**
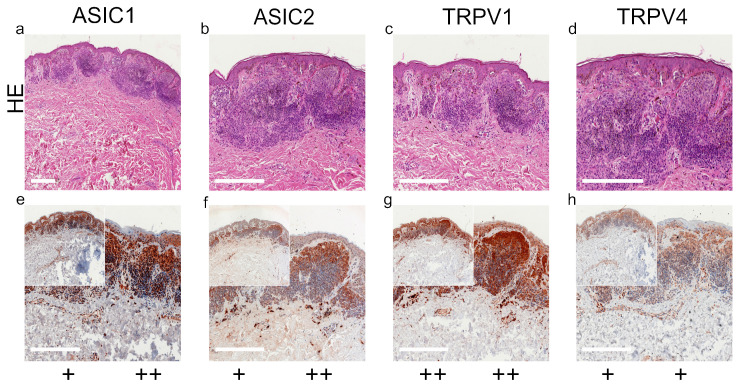
Immunohistochemistry of NCN. (**a**–**d**) H&E staining, (**e**–**h**) immunohistochemical staining, inserted smaller pictures show an overview. Scale bars represent 200 μm. (**a**–**h**) Patient 14. This NCN shows weak, positive expressions of ASIC1, ASIC2 and TRPV4 on the epidermal sections. TRPV1 is expressed strongly in the epidermis. Regarding the dermal area, ASIC1, ASIC2 and TRPV1 show strong expressions, whereas TRPV4 reveals a weak staining. It has to be mentioned that this NCN shows a decreasing expression in deeper dermal tumor tissue for all channels. For more stainings of other NCN, see Supplementary Figures S8–S11.

**Figure 4:**
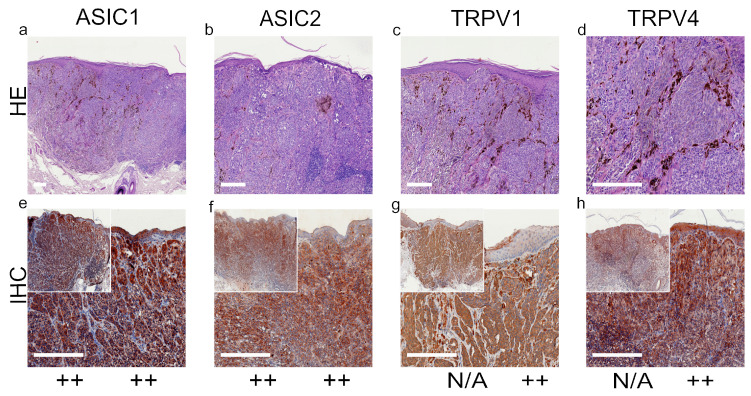
Immunohistochemistry of MM. Immunohistochemical staining for ASIC1, ASIC2, TRPV1 and TRPV4 in MM tissue. (**a**–**d**) Histochemical H&E staining, (**e**–**h**) immunohistochemical staining, inserted smaller pictures represent a two times larger perspective. Scale bars represent 200 μm. (**a**–**h**) Patient 30.

**Figure 5:**
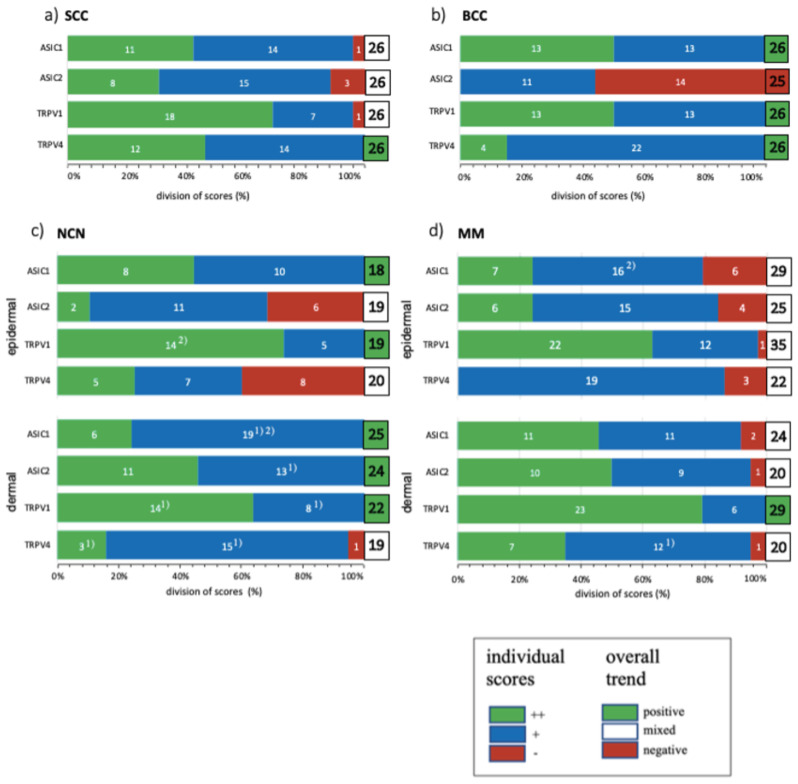
Summary of standard immunohistochemical and TMA score results for ASIC1, ASIC2, TRPV1 and TRPV4 on (**a**) SCC, (**b**) BCC, (**c**) NCN and (**d**) MM. ++/green bar: strong positive staining with >80% of cells positive and/or staining intensity is high; +/blue bar: 20–80% of cells show a weak positive/partial positive reaction; −/red bar: <20% of cells with weak staining (=negative reaction). NCN and MM are subdivided into epidermal and dermal portions. Numbers in bars represent the occurrence of the particular score. Superscript numbers give additional information: (1) some samples showed a decreasing expression in deeper dermal tumor tissue. (2) Single tumor cells are stained strong positive, others appear negative, resulting in an overall partial positive score (+). Overall trend is indicated by a green, white or red box with the number of samples investigated; green box: general positive reactions; white box: mixed reactions; red box: mainly negative reactions. For additional information on the individual TMA scores, see Supplementary Tables S2–S5.

*ASIC1*. A total of 42.4% of the SCC tissues were strong positive, 53.8% were weak positive and only 3.8% showed no expression (Figure 1a,e and Figure 5a, Supplementary Figures S1–S3 first column, Figure S16). Half of the BCC samples showed a strong expression, and the other half was weak positive (Figure 2a,e and Figure 5b, Supplementary Figures S4–S7 first column, Figure S16). The epidermal sections of NCN showed a strong positive staining in 44.4%, whereas in 55.5% we found a weak positive staining. The dermal sections were mainly stained weak positive (76%), the other 24% were strong positive (Figure 3a,e and Figure 5c, Supplementary Figures S8–S11 first column, Figure S16). We observed some nevi (marked with a ^1)^) with a decreasing expression in deeper dermal tumor tissue. In contrast, MM seem to not lose ASIC1 expression in deeper dermal portions. Epidermal melanoma tissues showed a weak positive staining in 55%, strong positive represented 24% and negative 21%. The dermal MM portions revealed 45.8% of strong positive and 45.8% of weak positive expression. Only 8.4% were negative (Figure 4a,e and Figure 5d, Supplementary Figures S12–S15 first column, Figure S16).

*ASIC2*. A total of 57.7% of SCCs showed a weak positive or partial expression of ASIC2. A total of 30.8% of SCC exhibited a strong positive expression, whereas 11.5% did not express ASIC2 (Figure 1b,f and Figure 5a, Supplementary Figures S1–S3 second column, Figure S16). In BCC, we observed no expression of ASIC2 in 56%, and 44% revealed a weak expression (Figure 2b,f and Figure 5b, Supplementary Figures S4–S7 second column, Figure S16). The epidermal portions of NCN showed mainly weak positive expression (57.9%); 10.5% of the samples were strong positive and 31.6% were negative. The dermal portions were strong positive in 45.8%, and weak positive in 54.2%. Just as with ASIC1 expression, we also observed a decreasing expression of ASIC2 in deeper dermal tumor tissue (Figure 3b,f and Figure 5c, Supplementary Figures S8–S11 second column, Figure S16). Concerning the epidermal parts of MM, the majority were weak positive (60%), whereas 24% were strong positive and 16% were negative. In dermal MM portions, half of our tissues expressed ASIC2 strongly (50%), 45% expressed it weakly and only 5% showed no expression (Figure 4b,f and Figure 5d, Supplementary Figures S12–S15 second column, Figure S16).

*TRPV1*. In SCC, the majority showed a strong positive expression (69%), 27% exhibited a weak expression and only 4% were negative (Figure 1c,g and Figure 5a, Supplementary Figures S1–S3 third column, Figure S16). In BCC, half of our samples were strong positive, the other half appeared weak positive (Figure 2c,g and Figure 5b, Supplementary Figures S4–S7 third column, Figure S16). The vast majority of the epidermal as well as the dermal parts of NCN appeared strong positive (epidermal: 73.6%, dermal: 63.6%), the rest was weak positive (epidermal: 26.4%, dermal: 36.4%) (Figure 3c,g and Figure 5c, Supplementary Figures S8–S11 third column, Figure S16). In MM, we also observed strong positive expression in the epidermis in 62.9% and in the dermis in even 79% (Figure 4c,g and Figure 5d, Supplementary Figures S12–S15 third column, Figure S16).

*TRPV4*. In SCC samples, 46.2% were strong positive for TRPV4, and the rest showed a weak positive expression (Figure 1d,h and Figure 5a, Supplementary Figures S1–S3 fourth column, Figure S16). In BCC, 15.4% revealed a strong positive expression, and 84.6% were weak positive (Figure 2d,h and Figure 5b, Supplementary Figures S4–S7 fourth column, Figure S16). In the epidermal parts of NCN, 40% of the tissue samples were negative, as compared to the majority of samples, with 35% weak positive and 25% strong positive staining. The dermal sections revealed 78.9% weak positive staining, 15.8% strong positive and 5.3% negative expression (Figure 3d,h and Figure 5c, Supplementary Figures S8–S11 fourth column, Figure S16). Concerning MM, we observed no strong positive staining of the epidermis, weak positive staining in 86.3% and no expression in 13.7%. However, the dermal portions of MM showed strong positive results in 35% of tissue samples, 60% were weak positive and only 5% negative (Figure 4d,h and Figure 5d, Supplementary Figures S12–S15 fourth column, Figure S16).

This MM shows a strong positive epidermal expression of ASIC1 and ASIC2. Concerning the dermis, ASIC1, ASIC2, TRPV1 and TRPV4 show significantly high expressions. For more stainings of other MM, see Supplementary Figures S12–S15.

## 3. Discussion

In our study, we investigated the expressions of ASIC1, ASIC2, TRPV1 and TRPV4 in common skin tumors, namely SCC, BCC, NCN and MM. Every tumor shows specific expression patterns of the ion channels.

Striking results were that BCCs are often negative for ASIC2, while nearly all SCCs express this marker. Epidermal MM sometimes seem to lack ASIC1 in contrast to NCN. Dermal portions of MM show strong expression of TRPV1 more frequently than dermal NCN portions. Some NCNs show a decreasing ASIC1/2 expression in deeper dermal tumor tissue, while MMs seem to not lose ASIC1/2 in deeper dermal portions.

### 3.1. ASIC1

Concerning the tumor tissues investigated in this study, ASIC1 is strongly expressed in SCC, BCC and in NCN in both epidermal and dermal portions. Epidermal and dermal MM varied in expression levels. Even though in the literature there is little information about the expression of ASIC1 in melanomas and NMSC, the role in cancer progression has been proven in other tissues. In malignant glioma ASIC1 plays a role in the growth and migration of the tumor cells [23]. Gupta et al. detected that ASIC1 contributes to breast cancer pathogenesis and that ASIC1 inhibitors lead to a significant reduction in tumor growth in mice [24]. Even in human lung adenocarcinoma cells (cell line A549) ASIC1s might be a prognostic marker [6]. Taking these considerations and our results together, ASIC1 might serve as a potential therapeutic target, but further functional studies are required to fully understand the role of ASIC1 in tumor progression.

### 3.2. ASIC2

ASIC2 shows a negative expression profile in BCC, whereas the dermal portion of NCN is strongly expressed. These inhomogeneous results mirror previous knowledge concerning ASIC2 in other tumors. ASIC2 being less expressed is consistent with findings by Berdiev et al., who investigated ASIC2 in malignant gliomas [23]. The authors found that ASIC2 is not expressed in the plasma membrane of glial cells, whereas ASIC1 is indeed expressed on these tumor cells, analogous to our findings in BCC. According to them, ASIC1 and ASIC2 are co-expressed in normal cells, and the lack of ASIC2 in tumor cells leads to a large inward cation current. Inhibiting this current reduces glioma growth and cell migration [25]. It remains to be investigated if these voltage-independent cation currents present in gliomas are also existent in BCC, making the inhibition of this conductance a potential therapeutic target. Our results regarding the positive expression of ASIC2 in dermal NCN are in accordance with findings by Zhou et al. They detected an up-regulation of ASIC2 in colorectal cancer, leading to increased cell proliferation, whereas a knockdown had the opposite effect [26].

### 3.3. TRPV1

In all of our investigated tumors, expression of TRPV1 was high. As mentioned before, TRPV1 is associated with the processes of inflammation and calcium signaling [16]. As both chronic inflammation as well as abnormal calcium signaling play a role in tumorigenesis, it seems plausible that TRPV1 is involved in tumor progression. Marincsák et al. detected a drastic elevated expression of TRPV1 in SCC of the human tongue and in precancerous lesions [27]. Additionally, in other head-and-neck SCC localized on the oral floor or the gingiva, TRPV1 expression is upregulated [28]. To investigate the effect of TRPV1 antagonists on skin tumor formation, Park et al. treated TRPV1 in keratinocytes with competitive antagonists (AMG-9810 and SB-705498) to potentially use TRPV1 as a pharmacological target, but they could not find skin tumor promotion in epidermal keratinocytes treated by the antagonists [29]. Research linking TRPV1 to carcinogenesis treatment needs to be further conducted, as the evidence from the literature seems controversial so far. Thus, even if we could show that TRPV1 shows a higher expression level in all our investigated tumors, further studies need to be conducted to better understand the exact role of TRPV1 in skin tumor formation to use it as a potential therapeutic target.

### 3.4. TRPV4

TRPV4 is overexpressed in SCC, and in BCC it also shows a positive expression profile. In MM it shows mixed reactions, and in the dermal portion of NCN negative expression is predominant. Previous studies reported that TRPV4 was involved in tumorigenesis in different kinds of cancers, such as in esophageal squamous cell carcinoma, where we can see an upregulation of TRPV4 [30]. Huang et al. activated TRPV4 in esophageal SCC, which resulted in cellular migration of the tumor cells [30]. Additionally, in gastric cancer TRPV4 is upregulated and is even associated with higher tumor invasion, lymph node metastasis and poor survival [31]. Contradictory to our results, another research group detected a downregulation of TRPV4 in specific NMSC, as Bowen’s disease (BD), solar keratosis (SK), and also BCC and SCC [15]. We cannot support these findings, as our stainings deliver positive and even higher expression of TRPV4 in SCC and BCC compared to normal keratinocytes. Based on this, it is plausible to speculate that the expression of TRPV4 (as well as the other channels) varies between patients, types and subtypes of cancer and micro- and macroenvironments.

In conclusion, ASIC1, ASIC2, TRPV1 and TRPV4 are expressed by most common skin tumors. However, there are some interesting expression patterns and differences, as noted above. Our findings need to be reinforced by a larger sample size, RNA expression analysis (e.g., RNAScope) and by functional studies that investigate the precise roles of the ion channels in tumor formation. This could potentially lead to drugs that target the investigated ion channels in order to manipulate the TMA and/or the cellular response towards the inside-out pH gradient in solid skin cancers.

## 4. Materials and Methods

For our study, we utilized five standard paraffin-embedded tissue samples and a further 20–30 tissue microarray (TMA) samples per tumor entity, which were provided by the dermatopathological laboratory of the Department of Dermatology, University Medical Center Regensburg. Staining of normal skin is shown in all figures as well as in Figure S17 separately. As positive controls, we assumed mouse cerebellum for ASIC1 and ASIC2, kidney for TRPV1 and pancreas for TRPV4 (Supplementary Figure S18). For negative controls, we used ovary for ASIC1, prostate for ASIC2, uterus for TRPV1 and liver for TRPV4 (Supplementary Figure S18).

General patient information is shown in Supplementary Table S1. The investigated tissue samples were from biopsies older than 10 years, hence free to use according to German legislation.

### 4.1. Immunohistochemistry

Paraffin-embedded and fixed tissues along with the positive and negative controls were sliced and fixed on object plates. To remove the paraffin, the specimen slides were heated for 30 min at 77 °C followed by a descending alcohol series at room temperature: 2 × Xylol, 2 × 100% ethanol, 2 × 96% ethanol, 2 × 70% ethanol, each for 5 min. To prevent false-positive results, the endogenous peroxidase was blocked with 3% hydrogen peroxide for 10 min and washed in distilled water. During the preceding steps, a citrate buffer at pH 6 was cooked for 20 min, so the tissue sections could be immediately boiled for 30 min (except ASIC2). After that, the slides were cooled on ice for 20 min and rested in phosphate-buffered saline (PBS) for 10 min. In order to prevent unspecific antibody binding, we blocked the tissue sections with a blocking solution (ZytoChemPlusHRP Kit/Rabbit, Zytomed Systems GmbH, Schwerte, Germany) for 10 min and washed in PBS again. Subsequently, the slides were incubated with the primary antibody in the specific concentration and rested overnight at 4 °C (ASIC1: 1:400, Thermofisher, ASIC1 Polyclonal Antibody, No. PA5-26278; ASIC2: 1:200, Thermofisher, ASIC2 Polyclonal Antibody, No. PA5-26222; TRPV1: 1:500, Thermofisher Polyclonal Antibody, No. PA5-34288; TRPV4: 1:300, Abcam, Anti-TRPV4 Antibody, No. ab219192). The following day, we washed the tissue sections three times with PBS and then incubated the slides with the biotinylated secondary antibody for 30 min. After washing them again three times in PBS, they were incubated with Streptavidin HRP conjugate for 30 min and then washed three times with PBS. In the end, the tissue sections were stained with AEC plus (Dako, No. K 3469). When the required staining of the positive-control occurred, the reaction was stopped by distilled water, and the same procedure was performed with tissue sections and negative controls. To counterstain the sections, we used Mayers Haemalm (Roth, No. T865.3).

The slides were scanned with PreciPoint M8, and the digital images were edited with ViewPoint online (PreciPoint, Freising, Bavaria). Finally, we assessed the staining of the sections by visual inspection, and we scored ++ for strong positive/positive reaction with >80% of cells positive and/or staining intensity is high, + for 20–80% of cells with a weak positive/partial positive reaction, and − for <20% of cells with weak staining (=negative reaction). The epidermis was used as a reference structure to determine scoring.

### 4.2. Tissue MiroArray (TMA)

By using TMA, we avoided experimental variability by staining multiple tissue samples simultaneously on a single slide. Thereby, we obtained representative tumor material from 20–30 samples per tumor type using the immunostaining protocol above. The range of our samples varied, as some samples did not show tumor cell nests on the sliced tissue and therefore could not be used for evaluation.

## Supplementary Materials

The following are available online at https://www.mdpi.com/article/10.3390/ijms22116024/s1, Figures S1–S3: Immunohistochemistry of SCC, Figures S4–S7: Immunohistochemistry of BCC, Figures S8–S11: Immunohistochemistry of NCN, Figures S12–S15: Immunohistochemistry of MM, Figure S16: Tissue microarray of SCC, BCC, NCN, MM and Met, Figure S17: normal skin stained for ASIC1, ASIC2, TRPV1 and TRPV4, Figure S18: Tissue controls for IHC/TMA staining, Table S1: patient data, Table S2: Scores of TMA-SCC, Table S3: Scores of TMA-BCC, Table S4: Scores of TMA-NCN, Table S5: Scores of TMA-MM.
