# Supplementary material for: Expression Profiles of ASIC1/2 and TRPV1/4 in Common Skin Tumors
Source: Int J Mol Sci. 2021 Jun 2;22(11):6024. doi: 10.3390/ijms22116024 (PMC8199644; doi:10.3390/ijms22116024)
Supplement: Supplementary file 1 [file ijms-22-06024-s001.zip › ijms-1227447-supplementary.pdf]

Supplementary Information:

Supplementary figures:

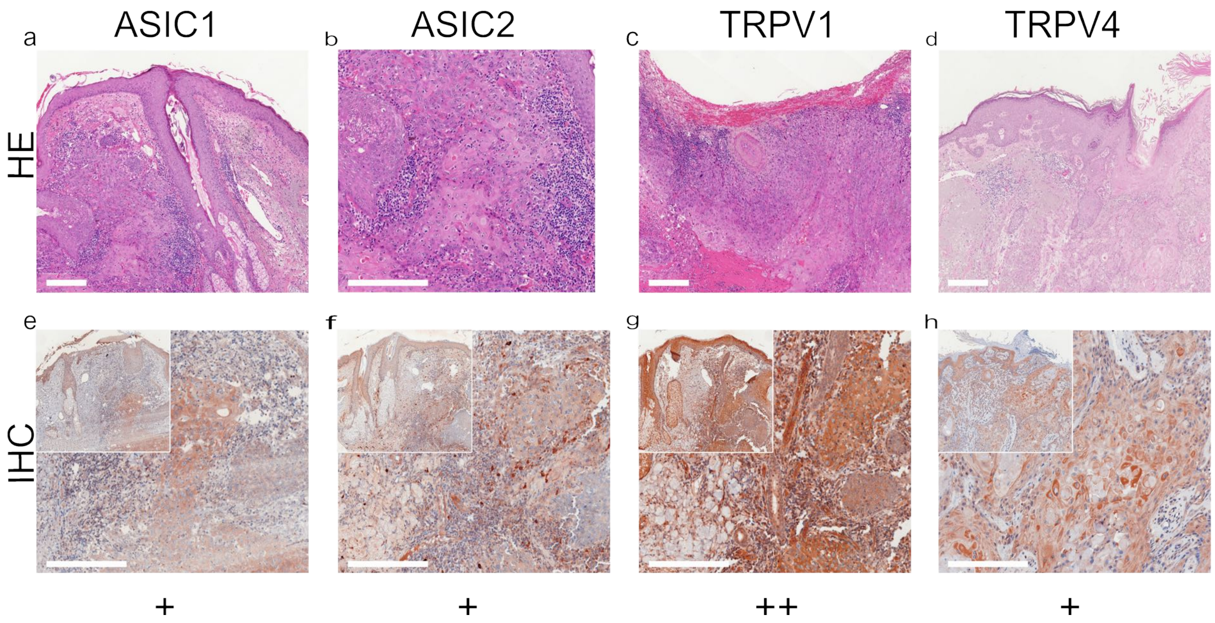

Figure S1 Immunohistochemistry of SCC. Immunohistochemical staining for ASIC1, ASIC2, TRPV1 and TRPV4 in SCC tissue. (a-d) H&E staining, (e-h) immunohistochemical staining, inserted smaller pictures represent a two times larger perspective. Scale bars represent 200 µm. a-c & e-g: patient 7; d, h: patient 1. This SCC shows a weak positive expression of ASIC1 and ASIC2. The expression of TRPV1 is high in tumor cells. For TRPV4, the representative SCC shows a weak expression, even if some tumor cells are stained intensely. For more stainings of other SCC, see supplementary figures S2-S3.

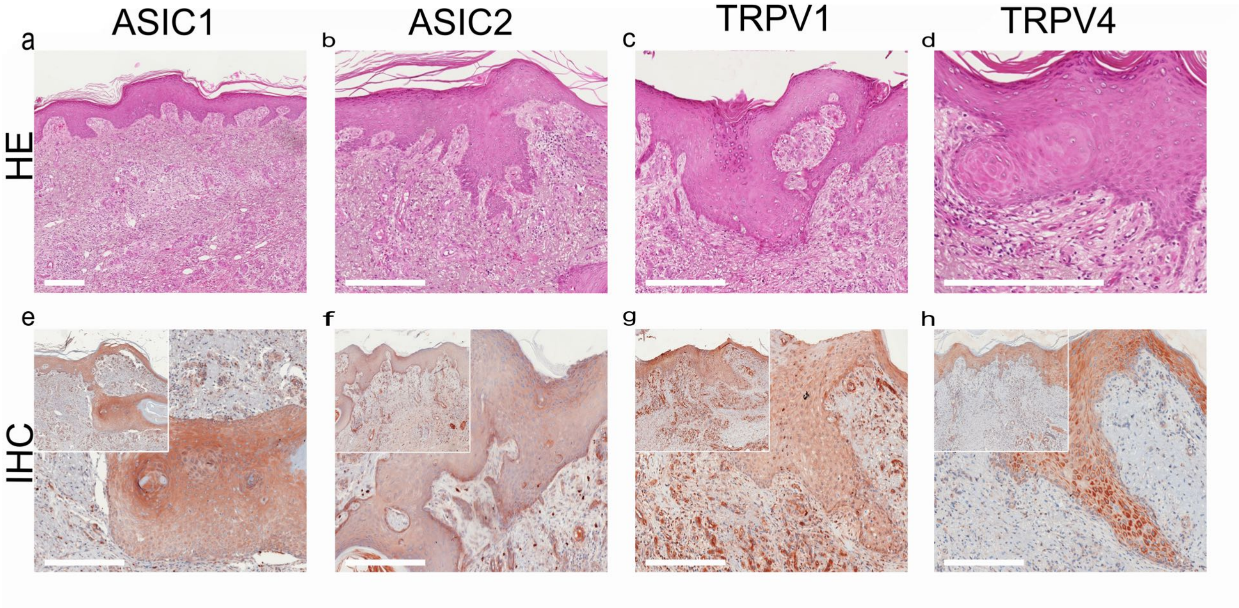

Figure S2. Immunohistochemistry of SCC. Immunohistochemical staining for ASIC1, ASIC2, TRPV1 and TRPV4 in SCC tissue. (a-d) histochemical H&E staining, (e-h) immunohistochemical staining, inserted smaller pictures represent a two times larger perspective. Scale bars represent 200 μm. a-h: patient 4. Scores in the bottom row were evaluated for ++: > 80 % of cells positive and / or staining intensity is high; +: 20-80 % of cells positive and staining weak or only partially strong; - : < 20 % cells with weak staining. This SCC shows a weak positive staining for ASIC1, ASIC2, TRPV1 and TRPV4. For more stainings of other SCC, see figures S1, S3.

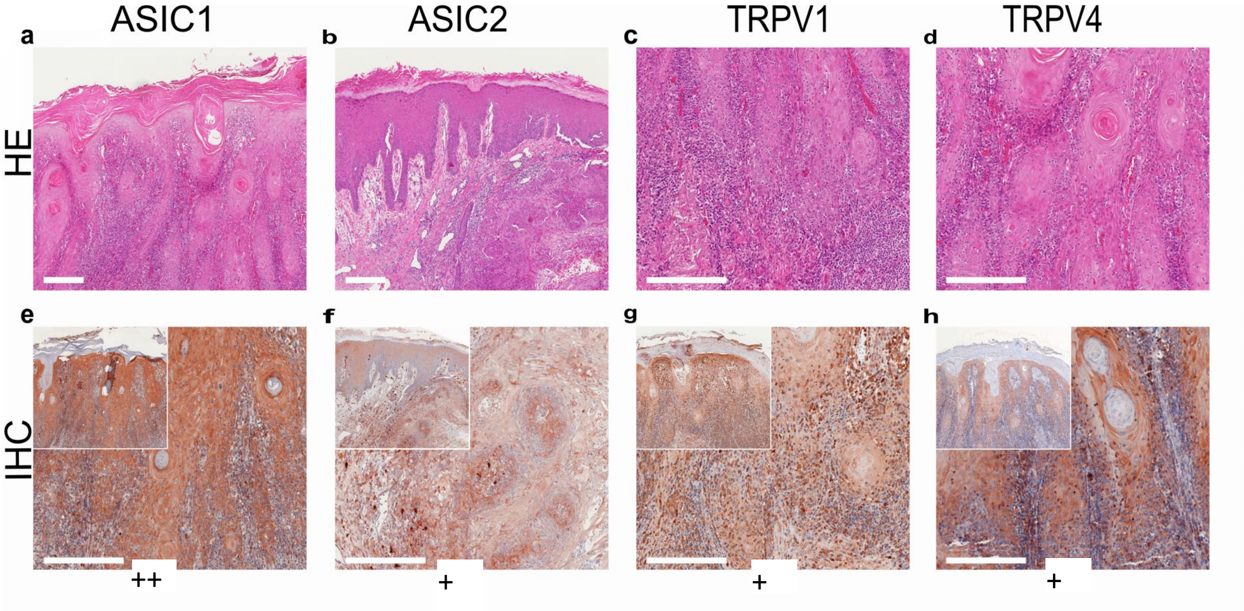

Figure S3. Immunohistochemistry of SCC. Immunohistochemical staining for ASIC1, ASIC2, TRPV1 and TRPV4 in SCC tissue. (a-d) histochemical H&E staining, (e-h) immunohistochemical staining, inserted smaller pictures represent a two times larger perspective. Scale bars represent 200 µm. a,c,d,e,g,h: patient 5; b,f: patient 2. Scores in the bottom row were evaluated for ++: > 80 % of cells positive and / or staining intensity is high; +: 20-80 % of cells positive and staining weak or only partially strong; - : < 20 % cells with weak staining. This SCC shows a strong positive expression of ASIC1. The expression of ASIC2, TRPV1 and TRPV4 is partial positive. For more stainings of other SCC, see figures S1-S2.

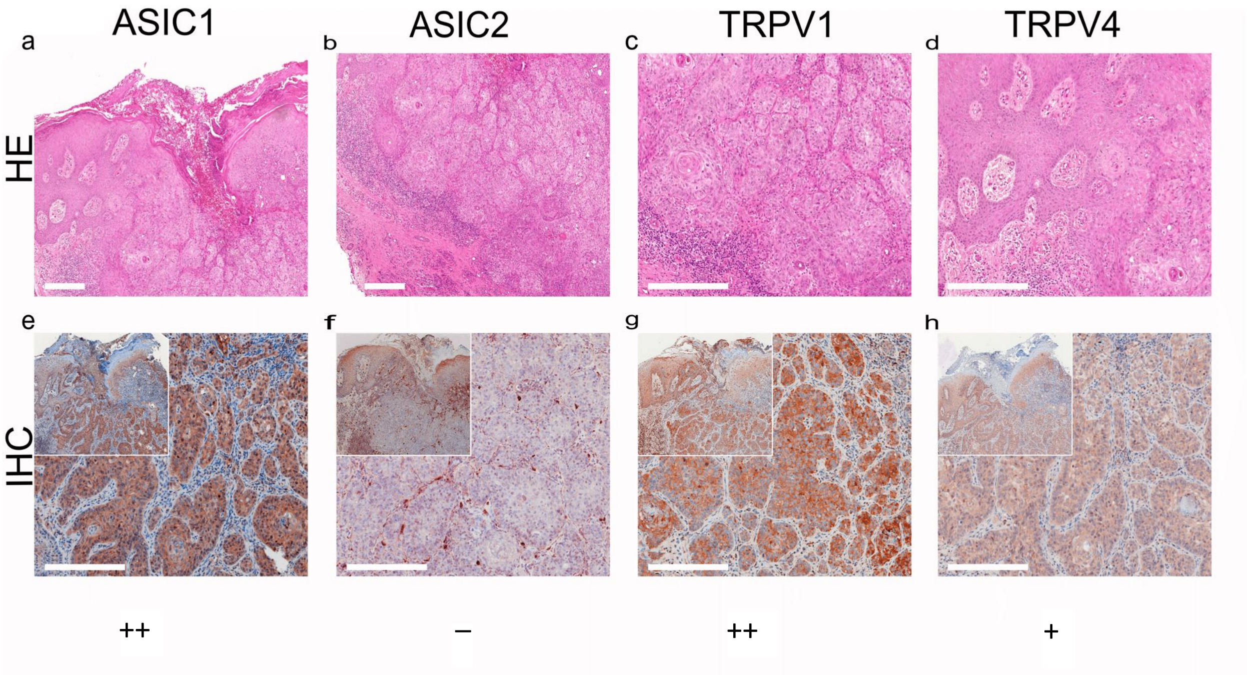

Figure S4. Immunohistochemistry of BCC. Immunohistochemical staining for ASIC1, ASIC2, TRPV1 and TRPV4 in BCC tissue. (a-d) histochemical H&E staining, (e-h) immunohistochemical staining, inserted smaller pictures represent a two times larger perspective. Scale bars represent 200 μm. a-h: patient 9. Scores in the bottom row were evaluated for ++: > 80 % of cells positive and / or staining intensity is high; +: 20-80 % of cells positive and staining weak or only partially strong; - : < 20 % cells with weak staining. This BCC shows a strong expression of ASIC1 and TRPV1. The expression of TRPV4 is weak positive, but this BCC shows no expression of ASIC2. For more stainings of other BCC, see figures S5-S7.

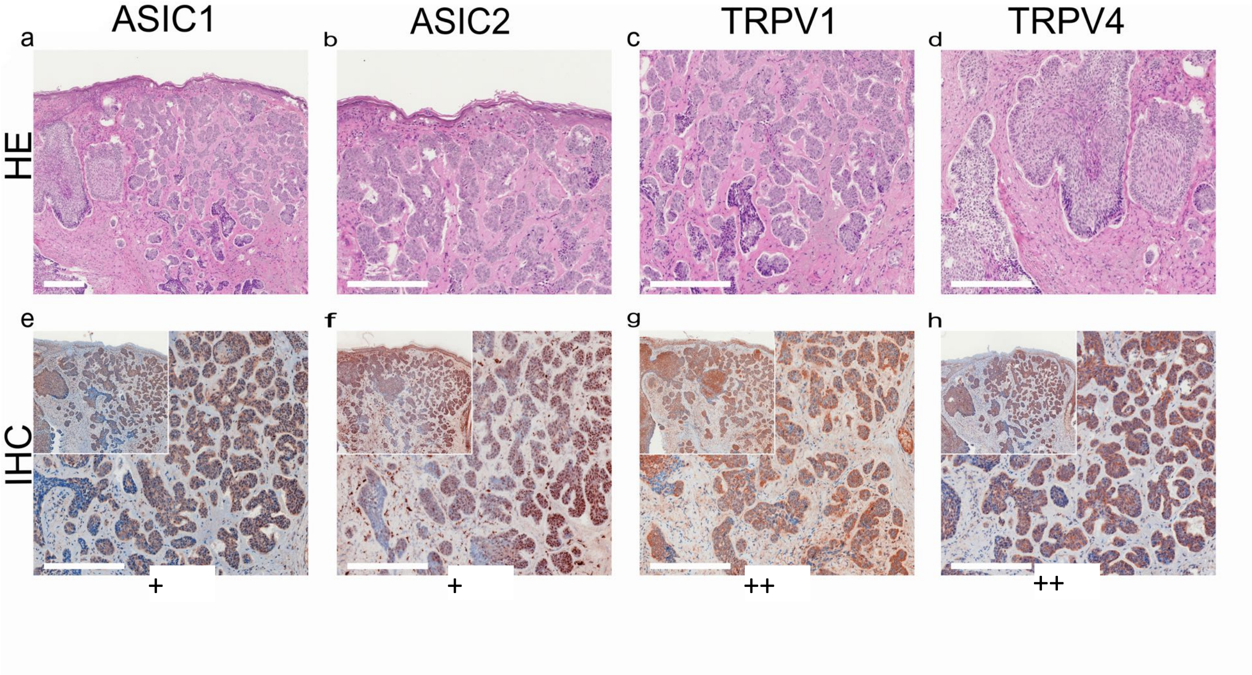

Figure S5. Immunohistochemistry of BCC. Immunohistochemical staining for ASIC1, ASIC2, TRPV1 and TRPV4 in BCC tissue. (a-d) histochemical H&E staining, (e-h) immunohistochemical staining, inserted smaller pictures represent a two times larger perspective. Scale bars represent 200 μm. a-h: patient 11. Scores in the bottom row were evaluated for ++: > 80 % of cells positive and / or staining intensity is high; +: 20-80 % of cells positive and staining weak or only partially strong; - : < 20 % cells with weak staining. This BCC shows a weak expression of ASIC1 and ASIC2. The expression of TRPV1 and TRPV4 is significantly increased in tumor cells.. For more stainings of other BCC, see figures S4,S6-S7.

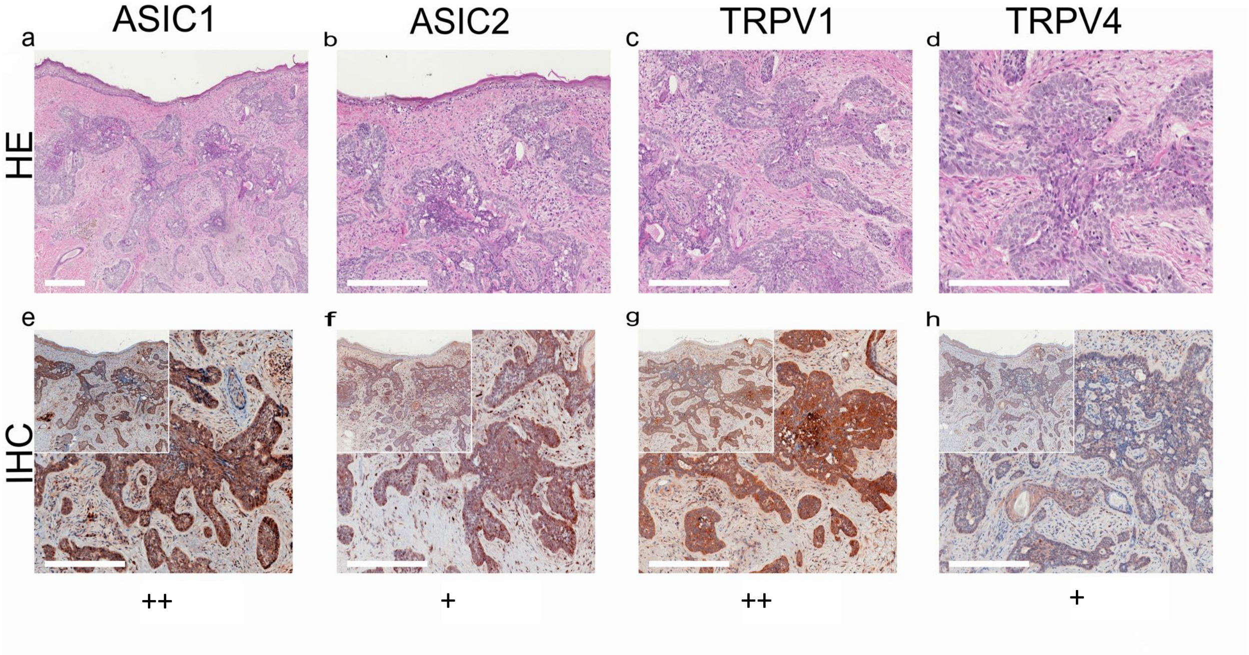

Figure S6. Immunohistochemistry of BCC. Immunohistochemical staining for ASIC1, ASIC2, TRPV1 and TRPV4 in BCC tissue. (a-d) histochemical H&E staining, (e-h) immunohistochemical staining, inserted smaller pictures represent a two times larger perspective. Scale bars represent 200 μm. a-h: patient 12. Scores in the bottom row were evaluated for ++: > 80 % of cells positive and / or staining intensity is high; +: 20-80 % of cells positive and staining weak or only partially strong; - : < 20 % cells with weak staining. ASIC1 and TRPV1 are strongly expressed on this BCC. The expression of ASIC2 and TRPV4 is weak positive. For more stainings of other BCC, see figures S4-S5, S7.

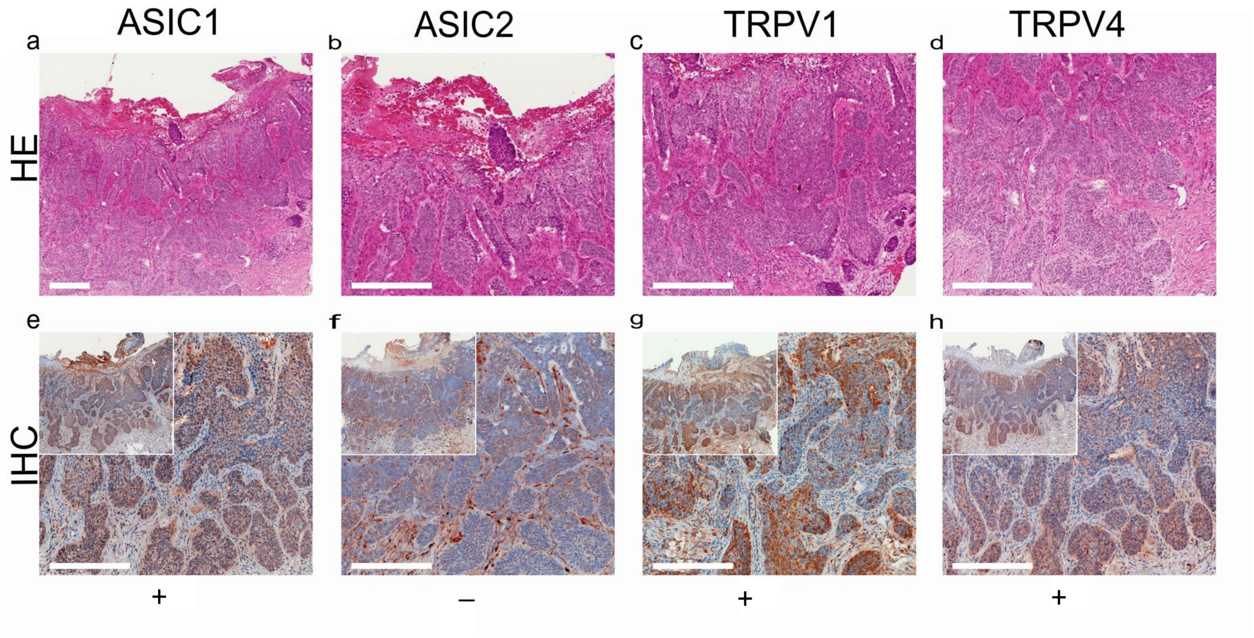

Figure S7. Immunohistochemistry of BCC. Immunohistochemical staining for ASIC1, ASIC2, TRPV1 and TRPV4 in BCC tissue. (a-d) histochemical H&E staining, (e-h) immunohistochemical staining, inserted smaller pictures represent a two times larger perspective. Scale bars represent 200 μm. a-h: patient 13. Scores in the bottom row were evaluated for ++: > 80 % of cells positive and / or staining intensity is high; +: 20-80 % of cells positive and staining weak or only partially strong; - : < 20 % cells with weak staining. This BCC shows no expression of ASIC2. The expression of ASIC1, TRPV1 and TRPV4 is weak positive. For more stainings of other BCC, see figures S4-S6.

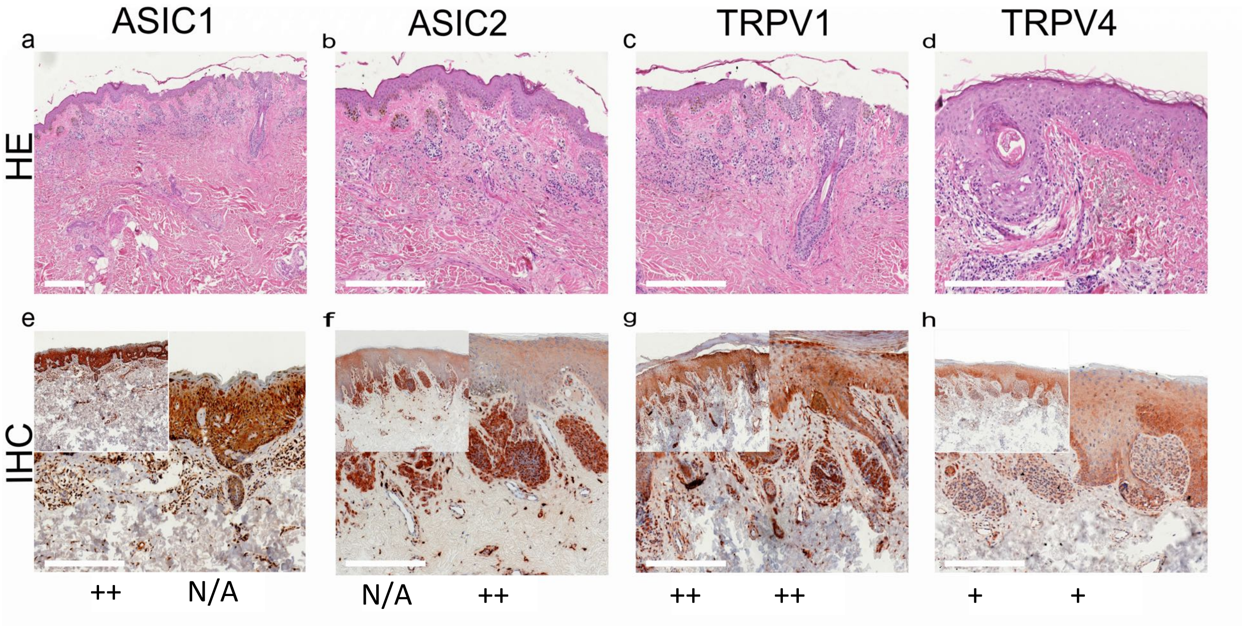

Figure S8. Immunohistochemistry of NCN. Immunohistochemical staining for ASIC1, ASIC2, TRPV1 and TRPV4 in NCN tissue. (a-d) histochemical H&E staining, (e-h) immunohistochemical staining, inserted smaller pictures represent a two times larger perspective. Scale bars represent 200 μm. b-d, f-h: patient 16; a, e: patient 20. Scores in the bottom row were evaluated for ++: > 80 % of cells positive and / or staining intensity is high; +: 20-80 % of cells positive and staining weak or only partially strong; - : < 20 % cells with weak staining for the epidermal (left score) and the dermal (right score) portion. This NCN shows a strong positive expression of ASIC1 and TRPV1 on the epidermal sections. TRPV4 is expressed weakly and ASIC2 cannot be assessed in this epidermal section, as we see no tumor cell nests (N/A). The same counts for the dermal part in ASIC1. The expression of ASIC2 and TRPV1 is significantly increased in dermal tumor cells. TRPV4 shows a weak dermal expression. For more stainings of other NCN, see figures S9-S11.

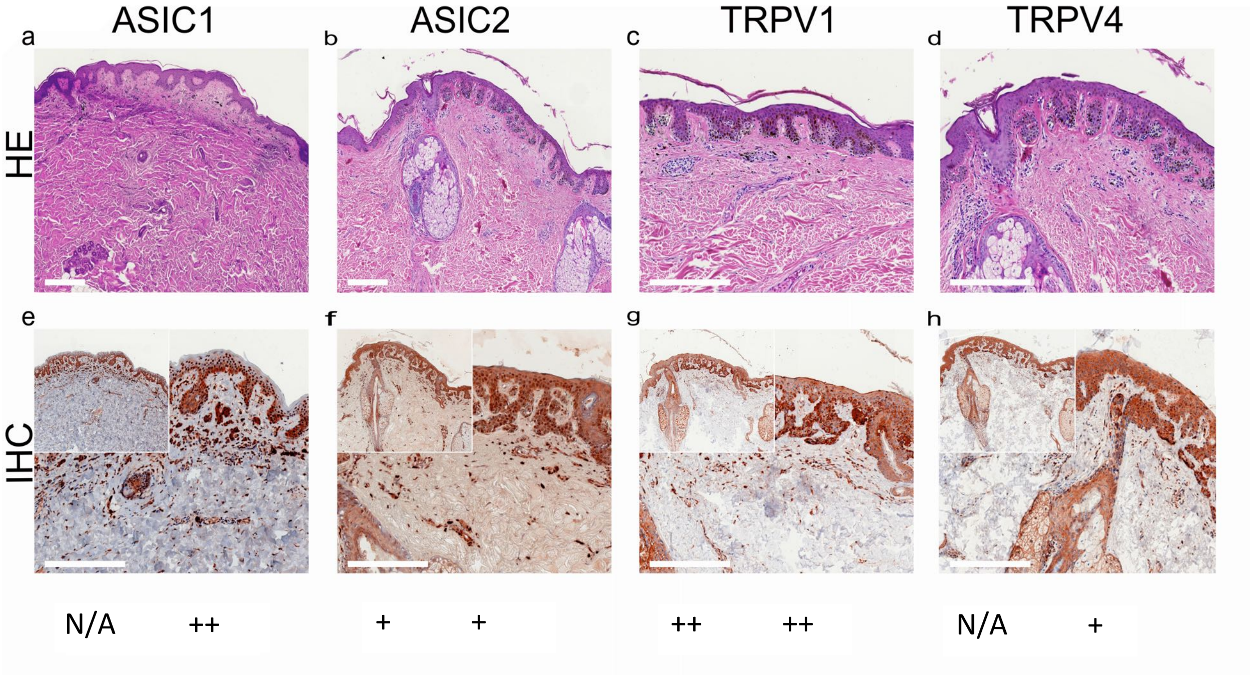

Figure S9. Immunohistochemistry of NCN. Immunohistochemical staining for ASIC1, ASIC2, TRPV1 and TRPV4 in NCN tissue. (a-d) histochemical H&E staining, (e-h) immunohistochemical staining, inserted smaller pictures represent a two times larger perspective. Scale bars represent 200 μm. a, e: patient 19; b-d & f-h: patient 17. Scores in the bottom row were evaluated for ++: > 80 % of cells positive and / or staining intensity is high; +: 20-80 % of cells positive and staining weak or only partially strong; - : < 20 % cells with weak staining for the epidermal (left score) and the dermal (right score) portion. This NCN shows a strong positive expression of TRPV1 on the epidermal sections and a weak positive expression of ASIC2. ASIC1 and TRPV1 cannot be assessed in this epidermal section, as we see no tumor cell nests (N/A). The expression of ASIC1 and TRPV1 is significantly increased in dermal tumor cells. ASIC2 and TRPV4 show a weak positive dermal expression. For more stainings of other NCN, see figures S8, S10-S11.

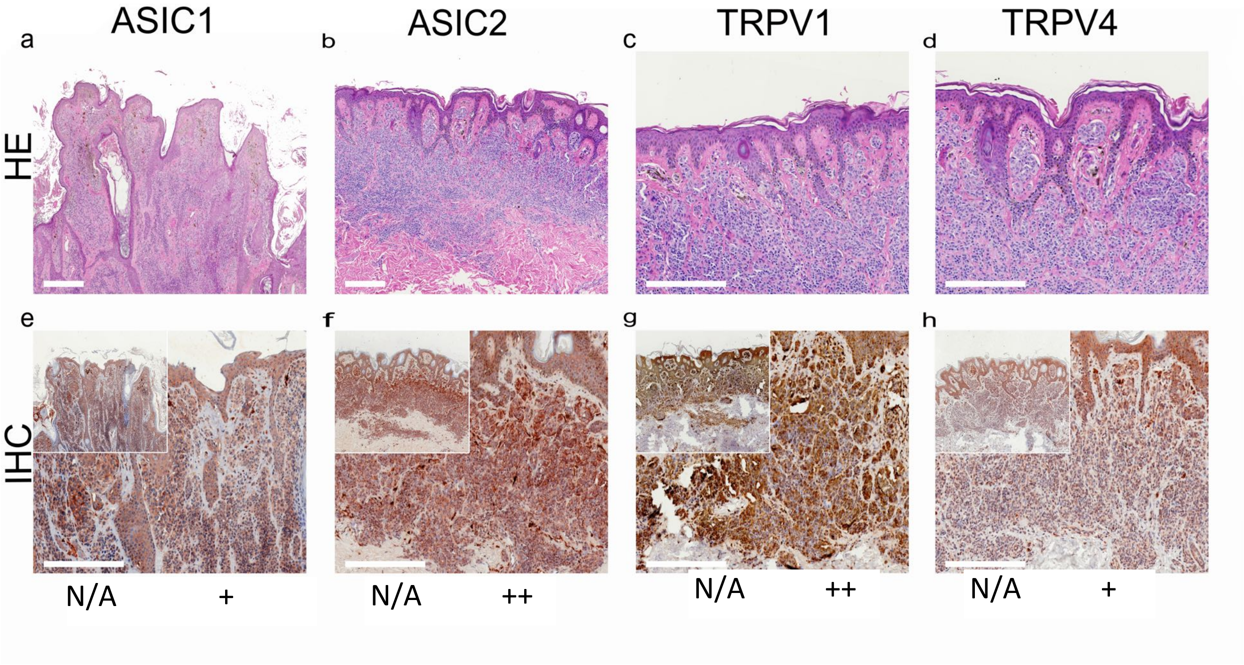

Figure S10. Immunohistochemistry of NCN. Immunohistochemical staining for ASIC1, ASIC2, TRPV1 and TRPV4 in NCN tissue. (a-d) histochemical H&E staining, (e-h) immunohistochemical staining, inserted smaller pictures represent a two times larger perspective. Scale bars represent 200 μm. b-d, f-h: patient 18; a, e: patient 21. Scores in the bottom row were evaluated for ++: > 80 % of cells positive and / or staining intensity is high; +: 20-80 % of cells positive and staining weak or only partially strong; - : < 20 % cells with weak staining for the epidermal (left score) and the dermal (right score) portion. In this NCN we can only assess the dermal portion, as we see no tumor cell nests in the epidermis (N/A). The expression of ASIC2 and TRPV1 is significantly increased in dermal tumor cells. ASIC1 and TRPV4 show a weak positive expression in the dermis. For more stainings of other NCN, see figures S8-S9, S11.

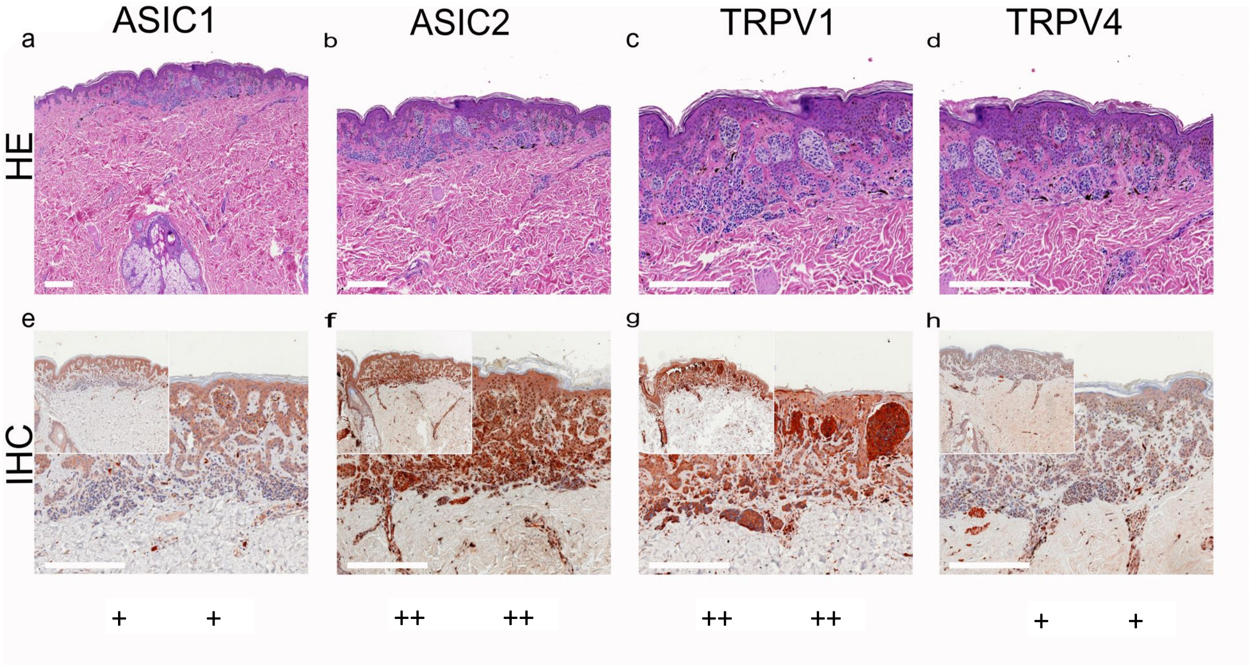

Figure S11. Immunohistochemistry of NCN. Immunohistochemical staining for ASIC1, ASIC2, TRPV1 and TRPV4 in NCN tissue. (a-d) histochemical H&E staining, (e-h) immunohistochemical staining, inserted smaller pictures represent a two times larger perspective. Scale bars represent 200 μm. a-h: patient 15. Scores in the bottom row were evaluated for ++: > 80 % of cells positive and / or staining intensity is high; +: 20-80 % of cells positive and staining weak or only partially strong; - : < 20 % cells with weak staining for the epidermal (left score) and the dermal (right score) portion. This NCN shows a strong positive expression of ASIC2 and TRPV1 on the epidermal sections and a weak positive expression of ASIC1 and TRPV4. Concerning the dermis, the expression of ASIC2 and TRPV1 is significantly increased in dermal tumor cells. ASIC1 and TRPV4 show a weak positive dermal expression. For more stainings of other NCN, see figures S8-S10.

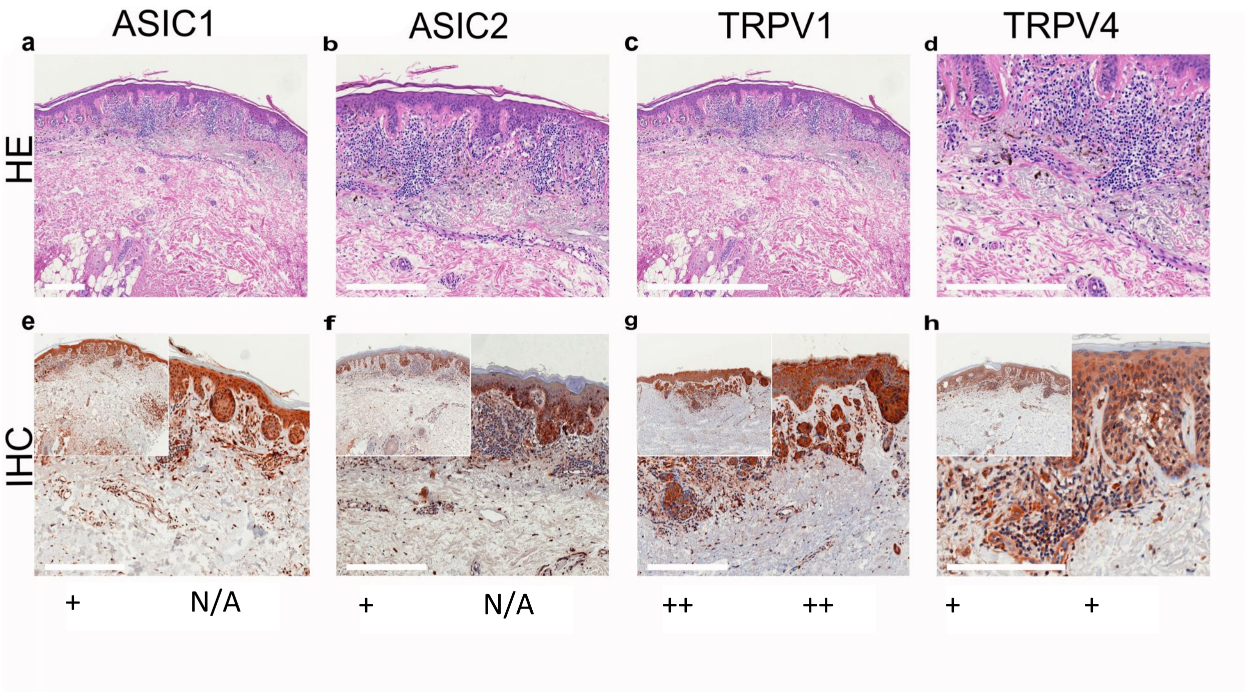

Figure S12. Immunohistochemistry of MM. Immunohistochemical staining for ASIC1, ASIC2, TRPV1 and TRPV4 in MM tissue. (a-d) histochemical H&E staining, (e-h) immunohistochemical staining, inserted smaller pictures represent a two times larger perspective. Scale bars represent 200 μm. a-h: patient 25. Scores in the bottom row were evaluated for ++: > 80 % of cells positive and / or staining intensity is high; +: 20-80 % of cells positive and staining weak or only partially strong; - : < 20 % cells with weak staining for the epidermal (left score) and the dermal (right score) portion.

This MM shows a weak positive expression of ASIC1, ASIC2 and TRPV4 on the epidermal sections and a strong positive expression of TRPV1. Concerning the dermis, the expression of TRPV1 is significantly increased in dermal tumor cells, TRPV4 shows a weak positive expression. In this MM we can not assess the dermal portion concerning ASIC1 and ASIC2, as we see no tumor cell nests. For more stainings of other MM, see figures S13-S15.

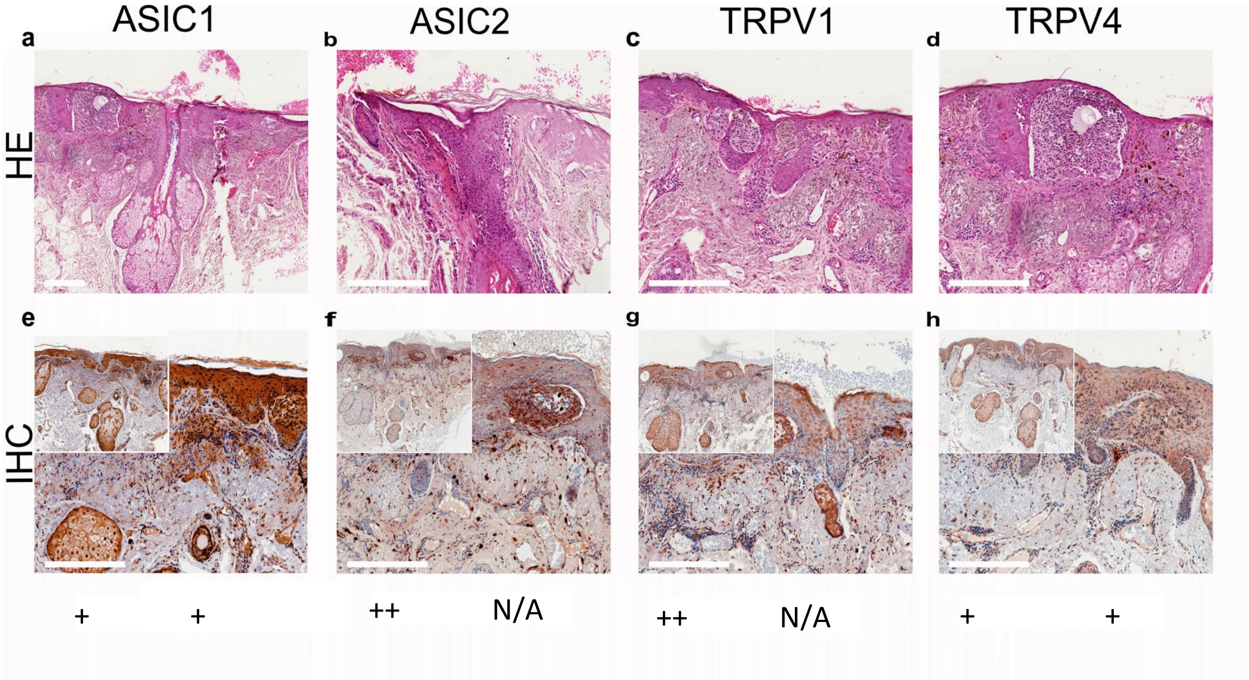

Figure S13. Immunohistochemistry of MM. Immunohistochemical staining for ASIC1, ASIC2, TRPV1 and TRPV4 in MM tissue. (a-d) histochemical H&E staining, (e-h) immunohistochemical staining, inserted smaller pictures represent a two times larger perspective. Scale bars represent 200 μm. a-h: patient 29. Scores in the bottom row were evaluated for ++: > 80 % of cells positive and / or staining intensity is high; +: 20-80 % of cells positive and staining weak or only partially strong; - : < 20 % cells with weak staining for the epidermal (left score) and the dermal (right score) portion.

This MM shows a weak positive epidermal expression of ASIC1 and TRPV4 and a strong positive expression of ASIC2 and TRPV1 in the epidermal sections. Concerning the dermis, ASIC1 and TRPV4 show a weak positive expression. In this MM we cannot assess the dermal portion concerning ASIC2 and TRPV1, as we see no tumor cell nests. For more stainings of other MM, see figures S12,S14-S15.

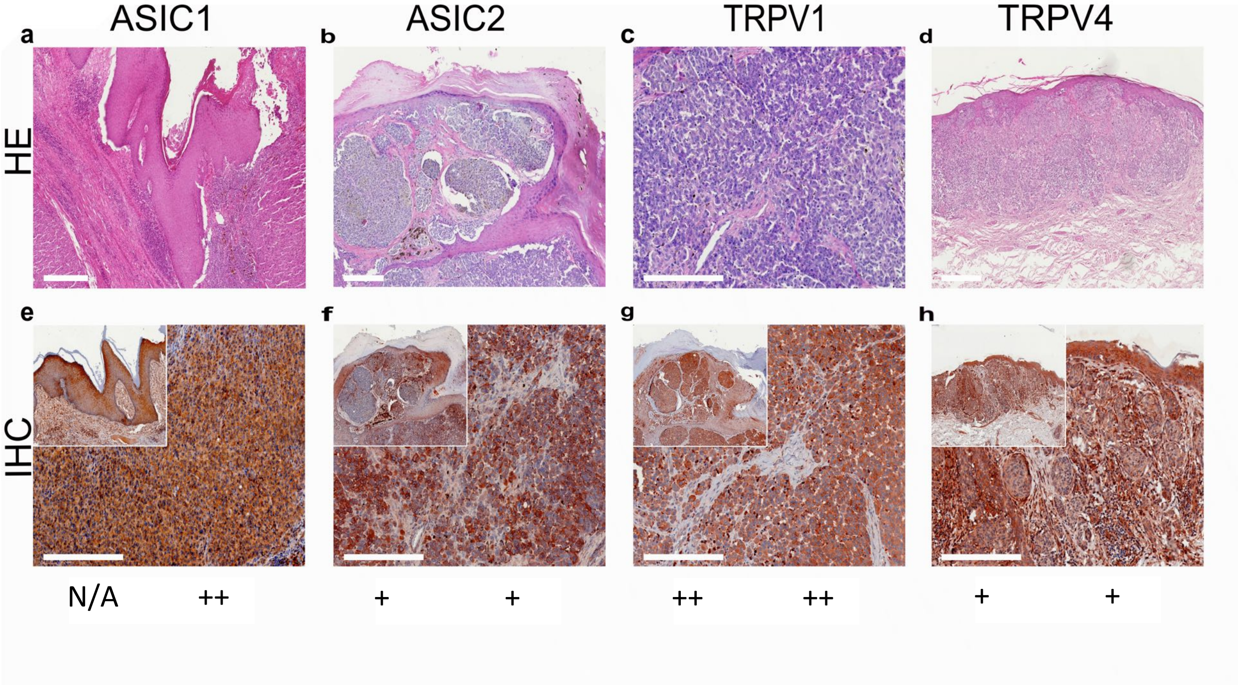

Figure S14. Immunohistochemistry of MM. Immunohistochemical staining for ASIC1, ASIC2, TRPV1 and TRPV4 in MM tissue. (a-d) histochemical H&E staining, (e-h) immunohistochemical staining, inserted smaller pictures represent a two times larger perspective. Scale bars represent 200 μm. a,e: patient 23, b,c,f,g: patient 22, d,h: patient 24 . Scores in the bottom row were evaluated for ++: > 80 % of cells positive and / or staining intensity is high; +: 20-80 % of cells positive and staining weak or only partially strong; - : < 20 % cells with weak staining for the epidermal (left score) and the dermal (right score) portion.

This MM shows a weak positive epidermal expression of ASIC2 and TRPV4 and a strong positive expression TRPV1 in the epidermal sections. In this MM we cannot assess the epidermal portion concerning ASIC1, as we see no tumor cell nests (N/A). In the dermis, ASIC1 and TRPV1 show a significantly increased expression. ASIC2 shows some strongly and weakly stained tumor cells giving an overall impression of a weak expression. TRPV4 is weakly expressed in the dermis. For more stainings of other MM, see figures S12-S13, S15.

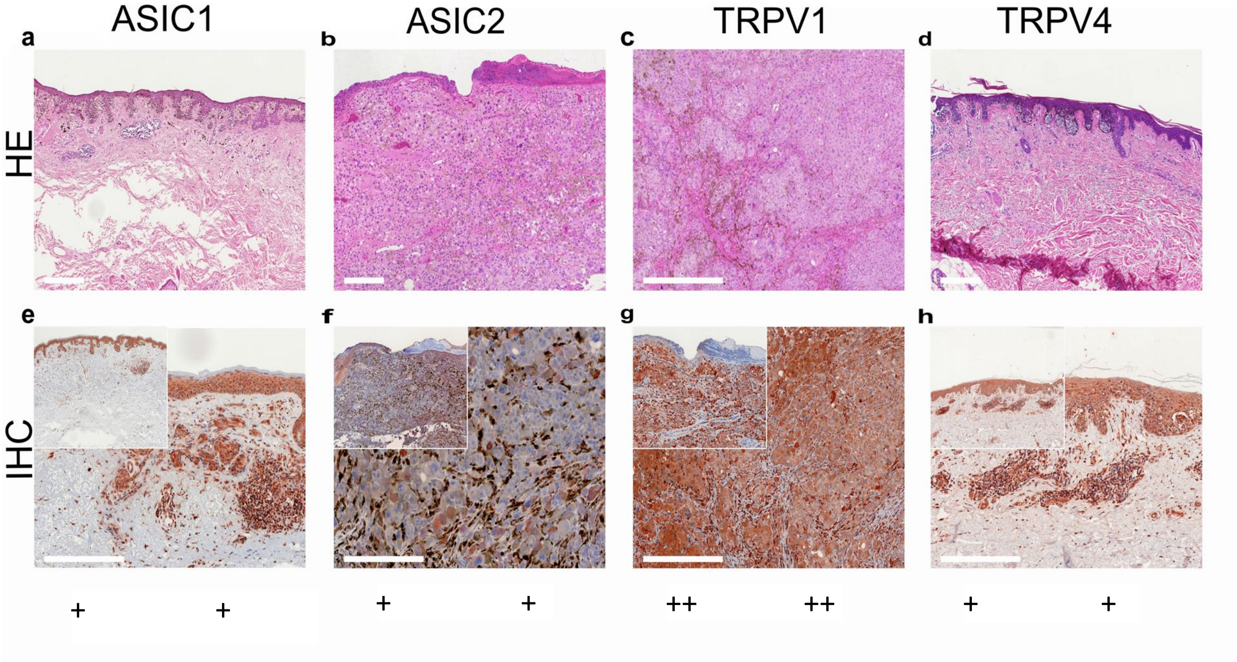

Figure S15. Immunohistochemistry of MM. Immunohistochemical staining for ASIC1, ASIC2, TRPV1 and TRPV4 in MM tissue. (a-d) histochemical H&E staining, (e-h) immunohistochemical staining, inserted smaller pictures represent a two times larger perspective. Scale bars represent 200 μm. a,d,e,h: patient 27; b,c,f,g: patient 26. Scores in the bottom row were evaluated for ++: > 80 % of cells positive and / or staining intensity is high; +: 20-80 % of cells positive and staining weak or only partially strong; - : < 20 % cells with weak staining for the epidermal (left score) and the dermal (right score) portion.

This MM shows a weak positive epidermal expression of ASIC1, ASIC2 and TRPV4 and a strong positive expression TRPV1 in the epidermal sections. Concerning the dermis, ASIC1, ASIC2 and TRPV4 show a weak positive expression. It has to be mentioned that ASIC2 shows tumor cells with a very strong staining and some hardly stained tumor cells giving an overall impression of a weak expression. TRPV1 is strongly expressed in the dermis. For more stainings of other MM, see figures S12-S14.

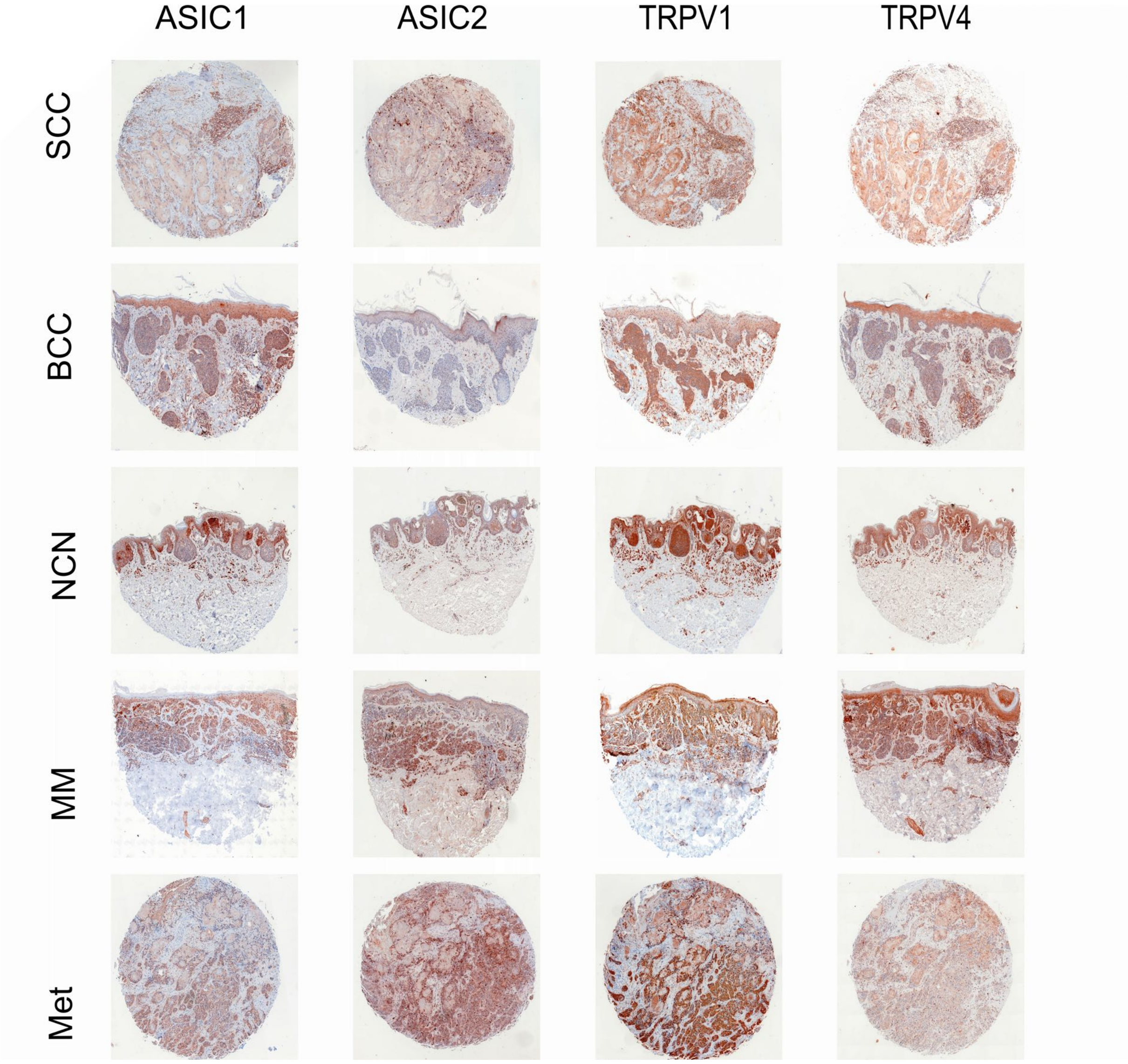

Figure S16: Tissue microarray of SCC, BCC, NCN, MM and Met. Selection of Immunohistochemical tissue microarray staining for ASIC1, ASIC2, TRPV1 and TRPV4.

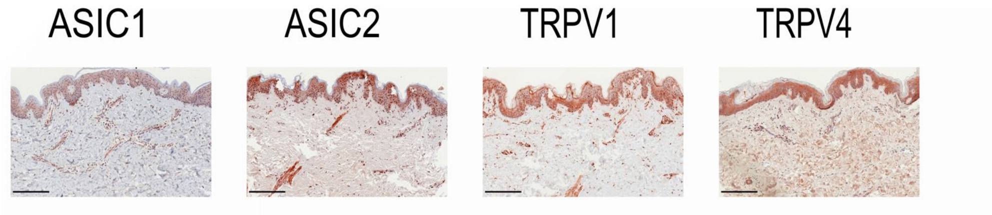

Figure S17: normal skin stained for ASIC1, ASIC2, TRPV1 and TRPV4.

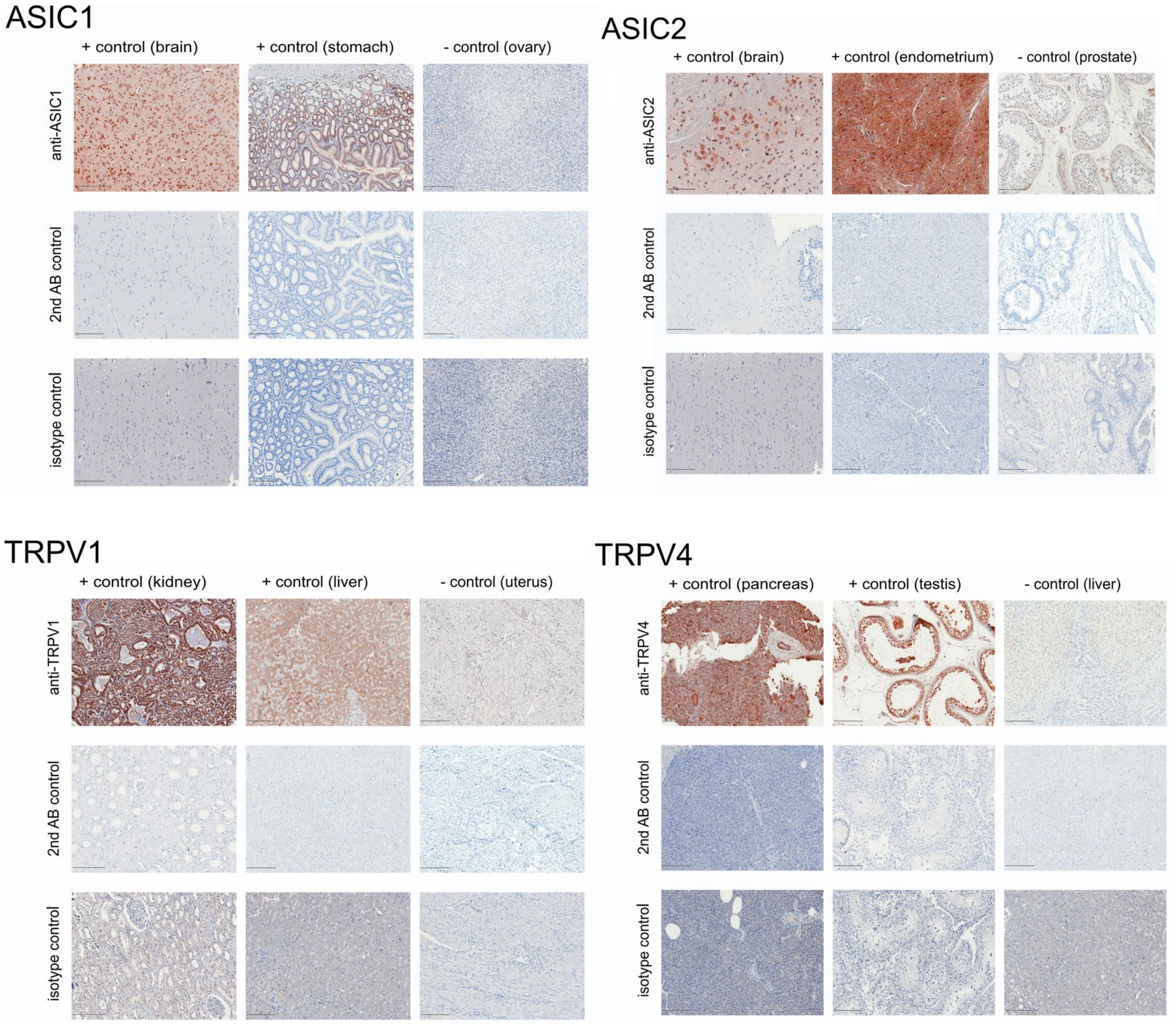

Figure S18: Tissue controls for IHC/TMA staining. IHC staining on positive and negative control tissues. We selected one positive control ("+ control) tissue according to the primary antibody datasheet and a second positive control and a negative control ("- control") for each cation channel (ASIC1/ASIC2/TRPV1/TRPV4) according to the database "The Human Protein Atlas" (www.proteinatlas.org). Control tissue were stained with the respective antibody, without primary antibody (secondary antibody control: "2nd AB control") and with the isotype control antibody. Scale bars correspond to 100 µm.

Supplementary tables:

Table S1. patient data: age and gender of the patients, the tissue samples were taken from.

patient	gender	age
1	♀	71
2	♂	59
3	♂	82
4	♀	87
5	♂	60
6	♂	75
7	♂	70
8	♀	45
9	♂	74
10	♀	95
11	♂	65
12	♀	72
13	♂	66
14	♂	40
15	♀	20
16	♂	40
17	♂	61
18	♀	11
19	♀	36
20	♀	36
21	♂	18
22	♂	62
23	♂	83
24	♀	36
25	♀	80
26	♀	43
27	♀	74
28	♂	75
29	♀	60
30	♂	89
31	♂	49
32	♀	80
33	♀	52
34	♂	40
35	♂	66
36	♂	71
37	♂	68
38	♀	36

39	♂	53
40	♂	16
41	♀	26
42	♀	48
43	♀	59
44	♀	21
45	♀	24
46	♀	71
47	♀	27
48	♀	28
49	♂	85
50	♀	46
51	♂	69
52	♂	68
53	♂	66
54	♀	44
55	♀	38
56	♀	54
57	♀	82
58	♂	67
59	♂	44
60	♀	72
61	♂	71
62	♀	78
63	♀	67
64	♂	44
65	♀	70
66	♂	60
67	♂	60
68	♀	63
69	♂	61
70	♀	78
71	♂	55
72	♀	38
73	♂	55
74	♀	54
75	♂	67
76	♂	44
77	♀	72
78	♂	71
79	♂	36
80	♂	71
81	♂	69

82	♀	78
83	♀	67
84	♂	44
85	♀	70
86	♂	87
87	♂	60
88	♂	60
89	♂	40
90	♂	72
91	♂	67
92	♂	59
93	♀	76
94	♀	79
95	♂	72
96	♂	68
97	♂	72
98	♀	45
99	♀	69
100	♂	68
101	♀	76
102	♀	79
103	♂	46
104	♀	56
105	♀	59
106	♂	65
107	♀	77
108	♀	53
109	♂	64
110	♀	67
111	♀	83
112	♂	65
113	♀	66
114	♂	63
115	♂	65
116	♂	81
117	♂	80
118	♂	80
119	♂	84
120	♂	74
121	♀	94
122	♀	51
123	♂	67
124	♀	89

125	♂	51
126	♂	48
127	♂	73
128	♂	63
129	♀	19
130	♂	69
131	♀	45
132	♀	93
133	♂	69
134	♀	45
135	♀	45
136	♂	83
137	♀	45
138	♂	83
139	♂	54
140	♂	77
141	♂	77
142	♂	64
143	♀	84
144	♂	78
145	♀	65
146	♀	75
147	♀	85
148	♀	83
149	♂	66
150	♂	69
151	♂	89
152	♀	55
153	♀	65
154	♀	71
155	♀	59
156	♀	76
157	♂	64
158	♀	69
159	♀	67
160	♀	63
161	♂	66
162	♀	50
163	♀	50
164	♀	19
165	♂	57
166	♀	19
167	♀	28

168	♀	43
169	♂	40
170	♂	64
171	♀	72
172	♂	44
173	♀	17
174	♂	66
175	♂	52
176	♀	67
177	♀	45
178	♂	25
179	♀	30
180	♀	8
181	♂	58
182	♂	37
183	♀	58
184	♂	36
185	♂	18
186	♀	33
187	♂	24
188	♂	64
189	♀	40
190	♂	37
191	♂	48
192	♀	37
193	♂	46
194	♂	52
195	♀	47

Table S2. Scores of TMA-SCC: Staining scores for ASIC1, ASIC2, TRPV1, TRPV4 on SCC tissues. ++: > 80 % of cells positive and / or staining intensity is high; +: 20-80 % of cells positive and staining weak or only partially strong; -: < 20 % cells with weak staining; N/A: not available, TMA_{SCC} 1-22: patient 53-79

TMA_{SCC}	ASIC1	ASIC2	TRPV1	TRPV4
1	+	+	+	+
2	++	++	+	++
3	++	+	++	+
4	+	+	++	+
5	+	++	++	+
6	-	-	+	+
7	+	+	++	++
8	++	+	++	+
9	+	+	++	+
10	+	+	++	+
11	++	++	++	++
12	+	++	++	++
13	+	+	++	+
14	++	+	++	++
15	+	++	+	++
16	+	+	++	++
17	++	+	++	++
18	+	++	++	++
19	++	+	++	++
20	+	-	-	+
21	++	++	++	++
22	++	++	++	++

Table S3. Scores of TMA-BCC: Staining scores for ASIC1, ASIC2, TRPV1, TRPV4 on BCC tissues. ++: > 80 % of cells positive and / or staining intensity is high; +: 20-80 % of cells positive and staining weak or only partially strong; -: < 20 % cells with weak staining; N/A: not available, TMA_{BCC} 1-22: patient 80-104

TMA_{BCC}	ASIC1	ASIC2	TRPV1	TRPV4
1	++	+	++	+
2	++	+	+	+
3	-	+	++	+
4	++	-	++	+
5	+	-	+	+
6	++	-	+	++
7	+	-	+	+
8	+	-	++	+
9	N/A	-	+	++
10	+	N/A	N/A	+
11	++	N/A	+	++
12	+	+	+	+
13	+	+	+	+
14	++	+	++	+
15	++	+	++	+
16	++	-	++	+
17	++	-	++	+
18	++	-	+	+
19	+	+	++	+
20	+	-	+	+
21	+	+	+	+
22	+	-	+	N/A

Table S4. Scores of TMA-NCN: Staining scores for ASIC1, ASIC2, TRPV1, TRPV4 on NCN tissues. ++: > 80 % of cells positive and / or staining intensity is high; +: 20-80 % of cells positive and staining weak or only partially strong; -: < 20 % cells with weak staining; N/A: not available, TMA_{NCN} 1-25: patient 105-128

TMA_{NCN}	ASIC1		ASIC2		TRPV1		TRPV4	
	epidermal	dermal	epidermal	dermal	epidermal	dermal	epidermal	dermal
1	+	+	N/A	+*[1]	N/A	++	-	+
2	N/A	+*[1]	N/A	+*[1]	N/A	+*[1]	N/A	+*[1]
3	N/A	+*[1]	N/A	+*[1]	N/A	+*[1]	N/A	+
4	+	N/A	-	N/A	++	N/A	-	N/A
5	++	+*[1]	-	+	+	+	-	+
6	++	++	+	++	N/A	++	++	++
7	N/A	N/A	-	N/A	++	N/A	-	N/A
8	+	+*[1]	-	+	+	+	+	+
9	+	+*[1]	+	++	++	+	+	+
10	N/A	N/A	N/A	+	N/A	N/A	N/A	N/A
11	N/A	N/A	N/A	+	N/A	N/A	N/A	N/A
12	N/A	N/A	N/A	N/A	+	N/A	++	N/A
13	++	+	+	+	++	++	-	++*[1]
14	+	+	+	++	++	++	-	++
15	+	N/A	+	++	+	++	-	N/A
16	+	+	+	+	++	++	+	+
17	++	N/A	N/A	N/A	N/A	N/A	N/A	N/A
18	++	++	+	++	++	++	++	+
19	++	+	+	++	++	+	++	-
20	N/A	+	-	+	+	++	N/A	N/A
21	++	+	+	++	++	+	+	+
22	N/A	+	N/A	N/A	N/A	N/A	N/A	N/A
23	N/A	+	N/A	N/A	N/A	N/A	N/A	N/A
24	N/A	++	N/A	N/A	N/A	N/A	N/A	N/A
25	N/A	+	N/A	N/A	N/A	N/A	N/A	N/A
26	+	+	N/A	N/A	N/A	N/A	N/A	N/A
27	N/A	+*[2]	N/A	N/A	N/A	N/A	N/A	N/A
28	N/A	++	N/A	N/A	N/A	N/A	N/A	N/A
29	N/A	N/A	-	+*[1]	N/A	N/A	N/A	N/A
30	N/A	N/A	++	+*[1]	N/A	N/A	N/A	N/A
31	N/A	N/A	N/A	N/A	N/A	++*[1]	N/A	N/A
32	N/A	N/A	N/A	N/A	++*[2]	+	N/A	N/A
33	N/A	N/A	N/A	N/A	N/A	N/A	++	+*[1]
34	N/A	N/A	N/A	N/A	N/A	N/A	-	+*[1]

*[1] expression level decreases with increasing tissue depth.

*[2] single tumor cells appear strong positive, others negative, giving the overall impression of weak positive expression.

Table S5. Scores of TMA-MM: Staining scores for ASIC1, ASIC2, TRPV1, TRPV4 on MM tissues. ++: > 80 % of cells positive and / or staining intensity is high; +: 20-80 % of cells positive and staining weak or only partially strong; - : < 20 % cells with weak staining; N/A: not available, TMA_{MM} 1-27: patient 1-27

TMA_{MM}	ASIC1		ASIC2		TRPV1		TRPV4	
	epidermal	dermal	epidermal	dermal	epidermal	dermal	epidermal	dermal
1	N/A	N/A	+	+	N/A	N/A	+	++
2	+	++	+	++	++	++	+	++
3	-	N/A	-	++	+	N/A	+	+
4	+	++	+	+	++	++	N/A	N/A
5	-	N/A	+	N/A	++	N/A	+	++
6	-	+	+	++	++	++	+	+
7	+	++	+	N/A	+	N/A	+	N/A
8	-	-	-	N/A	N/A	N/A	+	++
9	-	N/A	-	-	++	N/A	+	N/A
10	-	-	++	++	++	++	+	+
11	N/A	N/A	++	++	N/A	N/A	+	+
12	N/A	N/A	-	++	++	++	N/A	N/A
13	N/A	N/A	+	+	++	++	+	++
14	+	++	+	+	+	++	+	+
15	+	+	+	+	+	+	-	-
16	+	+	+	++	+	+	+	+
17	+	+	+	+	+	+	+	++
18	+	++	+	++	+	++	+	++
19	++	++	N/A	N/A	N/A	N/A	N/A	N/A
20	++	+	N/A	N/A	N/A	N/A	N/A	N/A
21	++	+	N/A	N/A	N/A	N/A	N/A	N/A
22	+	+	N/A	N/A	N/A	N/A	N/A	N/A
23	++	++	N/A	N/A	N/A	N/A	N/A	N/A
24	+	N/A	N/A	N/A	N/A	N/A	N/A	N/A
25	++	N/A	N/A	N/A	N/A	N/A	N/A	N/A
26	+*[1]	++	N/A	N/A	N/A	N/A	N/A	N/A
27	+	+	N/A	N/A	N/A	N/A	N/A	N/A
28	++	+	N/A	N/A	N/A	N/A	N/A	N/A
29	+	N/A	N/A	N/A	N/A	N/A	N/A	N/A
30	+	++	N/A	N/A	N/A	N/A	N/A	N/A
31	N/A	N/A	++	++	N/A	N/A	N/A	N/A
32	N/A	N/A	+	+	N/A	N/A	N/A	N/A
33	N/A	N/A	++	N/A	N/A	N/A	N/A	N/A
34	N/A	N/A	N/A	N/A	+	+	N/A	N/A
35	N/A	N/A	N/A	N/A	+	+	N/A	N/A
36	N/A	N/A	N/A	N/A	+	++	N/A	N/A
37	N/A	N/A	N/A	N/A	++	+	N/A	N/A
38	N/A	N/A	N/A	N/A	++	++	N/A	N/A
39	N/A	N/A	N/A	N/A	-	+	N/A	N/A

40	N/A	N/A	N/A	N/A	+	++	N/A	N/A
41	N/A	N/A	N/A	N/A	++	++	N/A	N/A
42	N/A	N/A	N/A	N/A	+	++	N/A	N/A
43	N/A	N/A	N/A	N/A	++	++	N/A	N/A
44	N/A	N/A	N/A	N/A	++	N/A	N/A	N/A
45	N/A	N/A	N/A	N/A	++	N/A	N/A	N/A
46	N/A	N/A	N/A	N/A	++	++	N/A	N/A
47	N/A	N/A	N/A	N/A	N/A	++	N/A	N/A
48	N/A	N/A	N/A	N/A	++	+	N/A	N/A
49	N/A	N/A	N/A	N/A	++	++	N/A	N/A
50	N/A	N/A	N/A	N/A	++	N/A	N/A	N/A
51	N/A	N/A	N/A	N/A	+	++	N/A	N/A
52	N/A	N/A	N/A	N/A	+	++	N/A	N/A
53	N/A	N/A	N/A	N/A	++	++	N/A	N/A
54	N/A	N/A	N/A	N/A	N/A	N/A	-	+
55	N/A	N/A	N/A	N/A	N/A	N/A	-	+*[2]
56	N/A	N/A	N/A	N/A	N/A	N/A	+	N/A

*[1] single tumor cells appear strong positive, others negative, giving the overall impression of weak positive expression.

*[2] expression level increases with increasing tissue depth.
